# Identify DNA-Binding Proteins Through the Extreme Gradient Boosting Algorithm

**DOI:** 10.3389/fgene.2021.821996

**Published:** 2022-01-28

**Authors:** Ziye Zhao, Wen Yang, Yixiao Zhai, Yingjian Liang, Yuming Zhao

**Affiliations:** ^1^ College of Information and Computer Engineering, Northeast Forestry University, Harbin, China; ^2^ International Medical Center, Shenzhen University General Hospital, Shenzhen, China; ^3^ Department of Obstetrics and Gynecology, The First Affiliated Hospital of Harbin Medical University, Harbin, China

**Keywords:** DNA-binding protein prediction, machine learning, feature extraction, dimensionality reduction, XGBoost model

## Abstract

The exploration of DNA-binding proteins (DBPs) is an important aspect of studying biological life activities. Research on life activities requires the support of scientific research results on DBPs. The decline in many life activities is closely related to DBPs. Generally, the detection method for identifying DBPs is achieved through biochemical experiments. This method is inefficient and requires considerable manpower, material resources and time. At present, several computational approaches have been developed to detect DBPs, among which machine learning (ML) algorithm-based computational techniques have shown excellent performance. In our experiments, our method uses fewer features and simpler recognition methods than other methods and simultaneously obtains satisfactory results. First, we use six feature extraction methods to extract sequence features from the same group of DBPs. Then, this feature information is spliced together, and the data are standardized. Finally, the extreme gradient boosting (XGBoost) model is used to construct an effective predictive model. Compared with other excellent methods, our proposed method has achieved better results. The accuracy achieved by our method is 78.26% for PDB2272 and 85.48% for PDB186. The accuracy of the experimental results achieved by our strategy is similar to that of previous detection methods.

## Introduction

Organisms contain many macromolecular substances, such as DNA and proteins, which contain the genetic information of organisms and are important components of all cells and tissues that make up an organism. To study the life activities of cells, it is necessary to study DNA and proteins and the interaction between them. Research on DBPs has an extremely important status and significance in related life sciences and plays an important role in DNA replication and recombination, virus infection and proliferation. It is necessary to study the combination of DNA and protein to study the gene expression of organisms at the molecular level. Researchers are paying increasing attention to DBP studies. DBPs are a kind of protein that binds to DNA, and it is critical to determine which of the numerous proteins can attach to DNA (Liu et al., 2019a; Li et al., 2019; Li et al., 2020) However, the traditional use of biochemical methods to find DBP consumes considerable time and money. Based on the above requirements and the development of computer science and ML(Zheng et al., 2019; Zheng et al., 2020; Wang et al., 2021a), relevant researchers have developed many detection methods based on ML algorithms in the hopes of improving the efficiency of detecting DBP and saving manpower and material resources.

ML is frequently utilized in the fields of computational biology (Jiang et al., 2013a; Cheng et al., 2019a; Liu et al., 2019b; Wang et al., 2019; Liu et al., 2020a; Tao et al., 2020a; Wang et al., 2020a; Zhang et al., 2020a; Zhao et al., 2020a; Zhu et al., 2020; Wang et al., 2021b; Wang et al., 2021c; Dao et al., 2021; Yu et al., 2021) to analyze brain disease (Liu et al., 2018a; Cheng et al., 2019b; Bi et al., 2020; Iqubal et al., 2020; Zhang et al., 2021a), lncRNA-miRNA interactions (Cheng et al., 2016; Liu et al., 2020b; Han et al., 2021), protein remote homology (Hong et al., 2020), protein functions (Wei et al., 2018a; Shen et al., 2019a; Shen et al., 2019b; Ding et al., 2019; Wang et al., 2020b; Shen et al., 2020; Tang et al., 2020; Wang et al., 2021d; Shang et al., 2021; Shao and Liu, 2021; Zhao et al., 2021), electron transport proteins (Ru et al., 2019), differential expression (Yu et al., 2020a; Zhao et al., 2020b; Zhai et al., 2020) and protein-protein interconnections (Ding et al., 2016a; Ding et al., 2016b; Yu et al., 2020b).

The protein sequence is very sizeable, and its number far exceeds the number of structures known to researchers (Zuo et al., 2017). Therefore, ML is used in various computer programs that predict DBP. The model IDNA-Prot|dis (Liu et al., 2014) was proposed by Liu et al. and is used to detect DBP based on the pseudo amino acid composition (PseAAC), and it can accurately extract the characteristics of DNA binding proteins. There are two models that use PseACC and physical-chemical distance transformation and support vector machine (SVM) algorithms, named PseDNA-Pro (Liu et al., 2015a) and iDNAPro-PseAAC (Liu et al., 2015b). Lin et al. developed the IDNA-Prot (Lin et al., 2011) prediction model based on the random forest (RF) algorithm through the PseACC feature. Kummar et al. developed two models based on RF and SVM classifiers called DNA-Prot (Kumar et al., 2009) and DNAbinder (Kumar et al., 2007). Dong et al. proposed the Kmer1+ACC (Liu et al., 2016) model based on the SVM algorithms Kmer composition and autocross covariance transformation. The position-specific scoring matrix (PSSM) can be obtained by calculating the protein sequence’s position frequency matrix, which has evolutionary information on the protein (Shao et al., 2021). The Local-DPP (Wei et al., 2017) uses the local pseudo position-specific scoring matrix (Pse-PSSM) and random forest algorithm to detect DBPs. Multiple kernel SVM is a DBP predictor from heuristically kernel alignment, and it is also named MKSVM-HKA (Ding et al., 2020a), which includes a variety of characteristics and was developed by Ding et al. The MSFBinder (Liu et al., 2018b) model proposed by Liu et al. is based on multiview features as well as classifiers. DPP-PseAAC (Rahman et al., 2018) is a model based on Chou’s general PseAAC, and it is used to detect DBPs. Methods have also been developed that combine multiscale features and deep neural networks to predict DBPs, such as MsDBP (Du et al., 2019).Adilina et al. (2019) analyzed protein sequence characteristics and implemented two different feature selection methods to build a DBP predictor.

In recent years, an increasing number of researchers have adopted complex feature extraction methods (Fu et al., 2020; Jin et al., 2021) and classification models to identify DBPs. It is critical to develop a method that uses as few DBP features as possible and includes a simple classification model while also ensuring a good ability to detect DPB. According to previous work, we proposed a DBP identification method based on the XGBoost model. First, several features were extracted from the protein sequence. Second, the features of these sequences were spliced. Third, the dimension of the data was standardized and reduced. Finally, the XGBoost model was used to detect DBPs. We have evaluated the effectiveness of our method on some benchmark data sets. Compared with some current experimental methods, our method achieves a better Matthew’s correlation coefficient (MCC), with a value of 0.713 for PDB186 and 0.5652 for PDB2272.

## Methods

**FIGURE 1:**
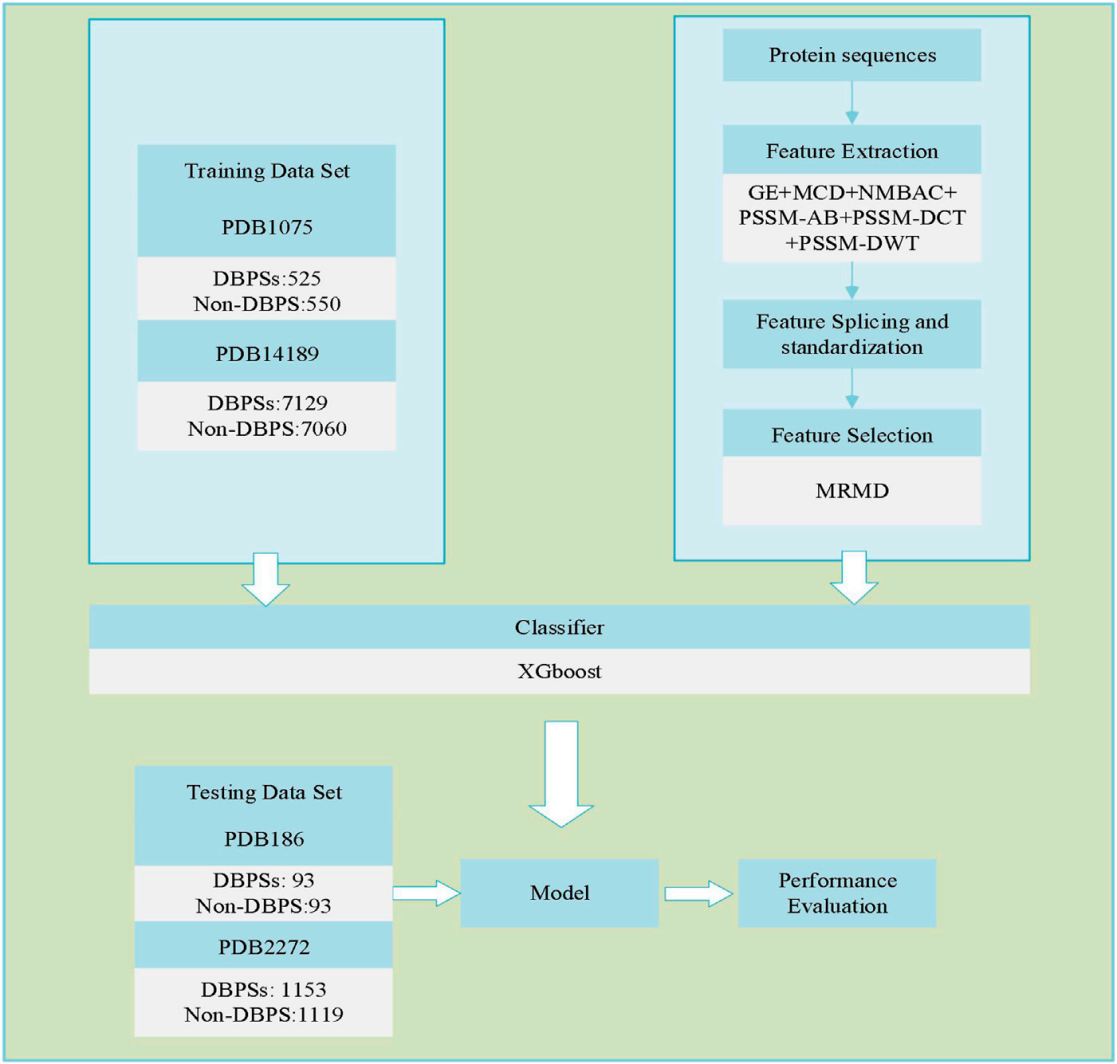
Process of predicting DBPs.

Identifying DBPs is a common dichotomy problem. First, we used six different feature extraction models for DBPs sequences to extract the corresponding sequence feature information. Then, the sequence feature information was spliced. Next, dimensionality reduction was performed on the spliced sequence feature information. Finally, the XGBoost model was utilized to identify DBPs. Figure 1 depicts the flowchart of our adopted technique.

### Extracting Features

To recognize DBPs, the corresponding features must be extracted. We adopt six feature extraction methods to obtain sequence information: global encoding, GE (Li et al., 2009); multi-scale continuous as well as discontinuous descriptor, MCD (You et al., 2014); normalized Moreau-Broto auto correlation, NMBAC (Ding et al., 2016b; Feng and Zhang, 2000); position specific scoring matrix-based average blocks, PSSM-AB (Jeong et al., 2011; Zhu et al., 2019); PSSM-based discrete cosine transform, PSSM-DCT (Huang et al., 2015); and PSSM-based discrete wavelet transform, PSSM-DWT (Nanni et al., 2012). The abovementioned feature extraction models are all well-known protein sequence extraction algorithm s and commonly used, which could be described in related works (Zou et al., 2021). Table 1 shows the feature dimensions derived by various feature extraction methods. After completing the above work, we used MATLAB to horizontally stitch together (Ding et al., 2020c; Ding et al., 2020d; Yang et al., 2021a) the features extracted from the same protein sequence using different feature extraction methods. The spliced features are represented by $Z^{*}$ . After splicing, the dimensions of PDB14189 and PDB2272 are 2692, and the dimensions of PDB1075 and PDB186 are 3092.

**TABLE 1:** Dimensional information about the features.

Model	Dimensionality
GE	150
MCD	882
MNBAC	200
PSSM-AB	200
PSSM-DCT	399
PSSM-DWT	1,040

### Standardize the Data

To make the data more standardized and unified and to strengthen the relationship between the characteristics of the data and the labels of the data, we use Z-score standardization to process the data.

Z-score standardization is defined as follows:

$$M*=\frac{Z_{i}^{*}-\bar{Z}}{\sigma }\;$$ (1A)

$$\bar{Z}=\frac{\sum_{i=0}^{N}Z_{i}^{*}}{N}$$ (1B)

$$\sigma =\sqrt{\frac{\sum_{i=0}^{N}{\left(Z_{i}^{*}-\bar{Z}\;\right)}^{2}}{N}}$$ (1C)

i=1,2,…,N (1D)

where N is the total number of samples and σ is the standard deviation.

The DBP sequence was processed in three stages: feature extraction, feature information splicing, and data standardization. Following the aforementioned three stages, we can obtain the sequence feature information $M^{*}$ .

### Dimensionality Reduction by Max-Relevance-Max-Distance

Zou et al. (Quan et al., 2016; Niu et al., 2020) developed a dimensionality reduction method in 2015 named Max-Relevance-Max-Distance (MRMD), and the user guide and complete runtime program can be obtained and downloaded from the following URL: https://github.com/heshida01/MRMD3.0. It judges data independence through a distance function and completes the dimensionality reduction operation in three steps (Tao et al., 2020b). It first evaluates each feature’s contribution to the classification and then quantifies each feature’s contribution to the classification. Second, the weights of different features are calculated for classification and the selected features are sorted accordingly. Third, the different numbers of features are filtered and classified and the results are recorded. We analyze and compare the results of the previous step to select the most effective group and use the sequence features chosen from this group as the result of dimensionality reduction.

The maximum correlation and the maximum distance are the main bases for the MRMD algorithm to judge the weight of each feature to the prediction result. The Pearson correlation coefficient can be used to quantify the degree of correlation between features and cases, and it can be calculated by the maximum relevance (MR).

The Pearson correlation coefficient is defined as follows:

$$\rho_{X,Y}=\frac{\mathrm{cov}\left(X,Y\right)}{\sigma_{X}\sigma_{Y}}$$ (2)

The *i*_*th*_ characteristic from the sequence and the category label to which those sequences belong make up the vectors X and Y. The maximum distance (MD) is used to assess feature redundancy. We calculate the three indices between characteristics in total.

$$\text{ED}\left(X,Y\right)=\sqrt{\sum_{i=0}^{N}{\left(x_{i}-\;y_{i}\right)}^{2}}\left(i=1,2,\ldots ,\text{N}\right)$$ (3A)

$$\cos\left(X,Y\right)=\frac{X\cdot Y}{\left\|X\right\|\left\|Y\right\|}$$ (3B)

$$TC\left(X,Y\right)=\frac{X\cdot Y}{{\left\|X\right\|}^{2}+{\left\|Y\right\|}^{2}-X\cdot Y}$$ (3C)

Equations 3A, E3B, E3C represent Euclidean distance, cosine similarity and Tanimoto coefficient, respectively. We can obtain the MD value by calculating the three indicators. Finally, the classification contribution value of each feature is calculated by combining MR and MD in a specific ratio.

After dimensionality reduction, the dimensions of PDB14189 and PDB2272 are 379, and the dimensions of PDB1075 and PDB186 are 1460.

Based on the three steps of feature extraction and splicing, data standardization and dimensionality reduction operations, we obtain the final sequence features.

### Extreme Gradient Boosting Algorithm

In 2011, Tianqi Chen and Carlos Guestrin (Chen and Guestrin, 2016) first proposed the XGBoost algorithm, or the extreme gradient boosting algorithm. It is a machine learning model that achieves a stronger learning effect by integrating multiple weak learners. The XGBoost model has many advantages, such as strong flexibility and scalability (Yang et al., 2021b; Zhang et al., 2021b).

Generally, most boosting tree models have difficulty implementing distributed training because when training n_th_ trees, they will be affected by the residuals of the first *n-1* trees and only use first-order derivative information. The XGBoost model is different. It performs a second-order Taylor expansion of the loss function and uses a variety of methods to prevent overfitting as much as possible. XGBoost can also automatically use the CPU’s multithreaded parallel computing to speed up the running speed. This feature represents a great advantage of XGBoost over other methods. XGBoost has improved significantly in terms of effect and performance.

The XGBoost algorithm is described in detail as follows:

$${\hat{y}}_{i}=\sum_{m=1}^{M}f_{m}\left(x_{i}\right),f_{m}\in F$$ (4)

where *M* is the number of trees and *F* represents the basic model of the trees.

The objective function is defined as follows:

$$L=\sum_{i}l\left({\hat{y}}_{i},y_{i}\right)+\sum_{m}\Omega \left(f_{m}\right)$$ (5)

The error between the predicted value and the true value is represented by the loss function *l*, and the regularized function Ω to prevent overfitting is defined as follows:

$$\Omega \left(f\right)=\gamma T+\frac{1}{2}\lambda {\left\|w\right\|}^{2}$$ (6)

where the weight and number of leaves of each tree are represented by w and *T*, respectively.

After performing the quadratic Taylor expansion on the objective function, the information gain generated after each split of the objective function can be expressed as follows:

$$Gain=\frac{1}{2}\left[\frac{{\left(\sum_{i\in I_{L}}g_{i}\right)}^{2}}{\sum_{i\in I_{L}}h_{i}+\lambda }+\frac{{\left(\sum_{i\in I_{R}}g_{i}\right)}^{2}}{\sum_{i\in I_{R}}h_{i}+\lambda }+\frac{{\left(\sum_{i\in I}g_{i}\right)}^{2}}{\sum_{i\in I}h_{i}+\lambda }\right]-\gamma$$ (7)

We can see that the split threshold γ is added to Eq. 7 to prevent overfitting and inhibit the overgrowth of the tree. Only when the information gain is greater than γ is the leaf node allowed to split. It can optimize the objective function at the same time because the tree is prepriced.

XGBoost also has the following two features:

1. Splitting stops when the threshold is greater than the weight of all samples on the leaf node too prevent the model from learning special training samples.
2. The features are randomly sampled when constructing each tree.

These features can prevent the XGBoost model from overfitting during the experiment.

## Experimental Results

In this chapter, we obtain experimental results through experiments on four benchmark data sets, evaluate our methods of identifying DBP and compare our experimental results with that of other methods.

### Data Sets

The four benchmark data sets are PDB1075, PDB186, PDB14189, and PDB2272. Liu et al. (2015a) and Lou et al. (2014) provided PDB1075 (training set) and PDB186 (independent testing set), respectively, and Du et al. (2019) provided PDB14189 (training set) and PDB2272 (independent testing set). These data sets are from the Protein Data Bank (PDB), and Table 2 shows the results of their detailed information.

**TABLE 2:** Basic information about four standard data sets.

Data sets	The number of negative	The number of positive	The total numbers
PDB14189	7,060	7,129	14,189
PDB1075	550	525	1,075
PDB2272	1,119	1,153	2,272
PDB186	93	93	186

### Measurement Standard

In this research, the following coefficients are used to evaluate our method: specificity (SP), sensitivity (SN), Matthew correlation coefficient (MCC), accuracy (ACC) and area under the ROC curve (AUC) (Jiang et al., 2013b; Wei et al., 2014; Wei et al., 2018a; Wei et al., 2018b; Cheng et al., 2018; Jin et al., 2019; Zhang et al., 2020b; Cheng et al., 2020; Liu et al., 2020c; Wang et al., 2020c; Guo et al., 2020; Huang et al., 2020; Wei et al., 2020; Zeng et al., 2020; Zhai et al., 2020). The calculation formulas for these coefficients are as follows:

$$Spec=\frac{TN}{TN+FP}$$ (8A)

$$SN=\frac{TP}{TP+FN}$$ (8B)

$$MCC=\frac{TP\times TN-FP\times FN}{\sqrt{\left(TP+FN\right)\times \left(TN+FP\right)\times \left(TP+FP\right)\times \left(TN+FN\right)}}$$ (8C)

$$ACC=\frac{TP+TN}{TP+TN+FP+FN}$$ (8D)

Among them, TN, TP, FP and FN reflect the values of true negatives, true positives, false positives, and false negatives, respectively.

### Performance Analysis

**FIGURE 2:**
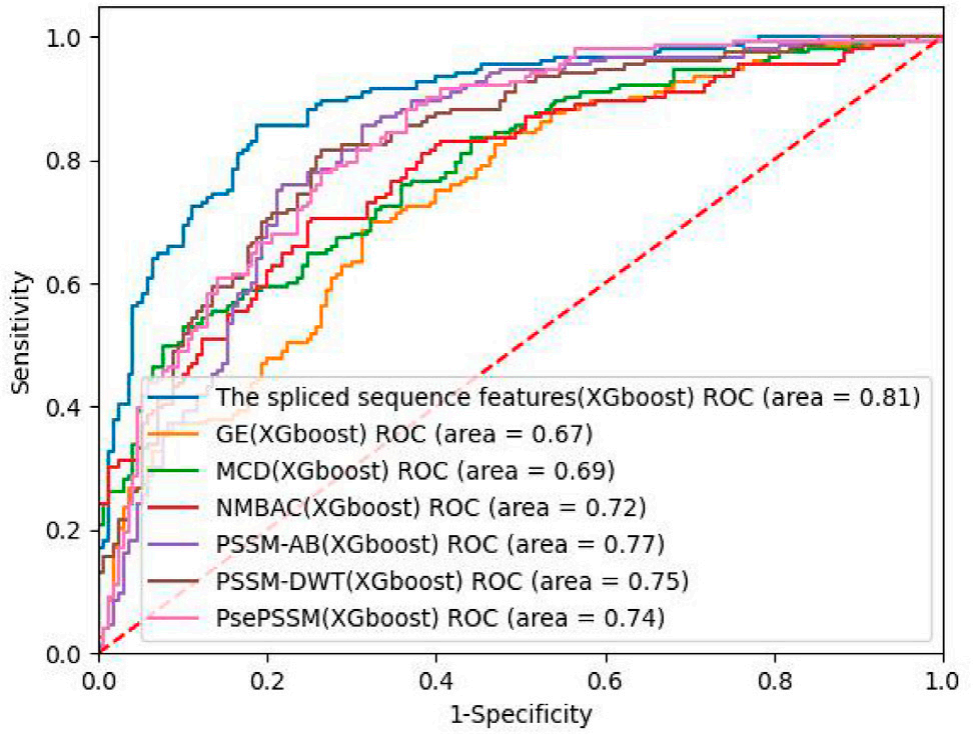
ROC curves of different feature extraction methods on PDB1075 data.

On the PDB 1075 data set, the performance of the spliced sequence features and single sequence features is evaluated by randomly extracting 30% of the data as a test set. Figure 2; Table 3 depict the experimental outcomes. PSSM-DWT (MCC: 0.4981) achieved better performance than other single sequence features. The spliced sequence features perform better than the single sequence feature on all parameters. The spliced sequence feature (ROC: 0.81) also gained the best ROC performance.

**TABLE 3:** Performance of PDB1075 using different feature extraction methods in XGBoost.

Model name	Feature extraction method	ACC (%)	SN (%)	MCC	Spec (%)
	GE	66.87	71.17	0.3342	62.09
	MCD	69.04	70.00	0.3975	67.97
	NMBAC	72.14	75.29	0.4404	68.62
XGboost	PSSM-AB	76.47	75.29	0.5300	77.77
	PSSM-Pse	74.30	75.88	0.4845	72.54
	PSSM-DWT	74.92	74.70	0.4981	75.16
	The spliced sequence feature	**81.42**	**84.11**	**0.6272**	**78.43**

Bold indicates that their experimental results are the best and the experimental values are the highest.

### Independent Data Set of PDB186

In this experiment, different sequence features have different prediction performances. We use PDB1075 as the training set and PDB186 as the test set to evaluate our experimental method and compared the experimental findings of our approach to those of 13 other methods. Table 4 clearly shows the complete experimental outcomes.

**TABLE 4:** Comparison between the XGBoost model and other methods on the PDB186 data set.

Models	ACC (%)	SN (%)	Spec (%)	MCC
IDNA-Prot|dis	72.0	79.5	64.5	0.445
IDNA-Prot	67.2	67.7	66.7	0.344
DNA-Prot	61.8	69.9	53.8	0.240
DNAbinder	60.8	57.0	64.5	0.216
DBPPre	76.9	79.6	74.2	0.538
IDNAPro-PseAAC	71.5	82.8	60.2	0.442
Kmerl + ACC	71.0	82.8	59.1	0.431
Local-DPP	79.0	92.5	65.6	0.625
DPP-PseAAC	77.4	83.0	70.9	0.550
MSFBinder	79.6	93.6	65.6	0.616
MsDBP	80.1	86.0	74.2	0.606
MKSVM-HKA	81.2	94.6	67.7	0.648
Adilina’s work	82.3	**95.0**	69.9	0.670
XGboost	**85.48**	90.3	**80.6**	**0.713**

Bold indicates that their experimental results are the best and the experimental values are the highest.

^a^The experimental results of other methods come from (Wei et al., 2017).

The MCC values of the five methods are all above 0.6 for MSDBP, MSFBinder, Local-DPP MKSVM-HKA, and Adilina’s work (0.606, 0.616, 0.625, 0.648 and 0.670, respectively). Thus, these methods have excellent performance. Although Adilina’s work (SN: 95.0%) performs best in terms of the value of SN, the results of XGBoost achieve optimal ACC (85.48%), MCC (0.713) and Spec (80.6%). On PDB1075 and PDB186, XGBoost outperforms the other methods.

### Independent Data Set of PDB2272

Du et al. (2019) removed proteins in PDB2272 that shared more than 40% of their sequence with PDB14189 to avoid homology bias between the two data sets. We conducted experiments on Du’s data set to verify the performance of the XGBoost model. PDB14189 is the training set, and PDB2272 is the test set. We independently tested XGBoost on PDB2272, used PDB14189 as the training set and compared it with five other classification methods. The detailed experimental results can be seen in Table 5. The results clearly show that XGBoost achieves the best ACC, MCC and Spec values of 78.26%, 0.5652 and 76.05%, respectively, compared with the other methods. For PDB2272, XGBoost presents a superior performance relative to the other classification methods.

**TABLE 5:** Experimental findings for the independent data set PDB2272 using the XGBoost algorithm and other models.

Methods	ACC (%)	MCC	SN (%)	Spec (%)
MK-FSVM-SVDD	76.12	0.5476	**91.50**	60.41
DPP-PseAAC	58.10	0.1625	56.63	59.61
PseDNA-Pro	61.88	0.2430	75.28	48.08
MK-SVM	75.00	0.5264	91.41	58.09
MsDBP	66.99	0.3397	70.69	63.18
XGboost	**78.26**	**0.5652**	80.39	**76.05**

Bold indicates that their experimental results are the best and the experimental values are the highest.

^a^The experimental results of other methods come from (Du et al., 2019; Zou et al., 2021).

### Experimental Results With PDB2272 and PDB186 as Test Set

We combined PDB14189 and PDB1075 as the training set, and combined PDB2272 and PDB186 as the test set. After normalization and dimensionality reduction operations, we got an accuracy of 79.09% and the MCC value was 0.5818. It can be seen that this result is between the previous two experimental results.

## Discussion and Conclusion

This paper proposes a method of predicting DBPs using the XGBoost algorithm and by splicing sequence feature information. The final sequence feature is built from multiple sequence features and spliced by MATLAB. To make the data more standardized and strengthen the relationship between data characteristics and data tags, the data are processed using Z-Score standardization. During the experiment, we used MRMD to reduce the dimensionality of the data and thus reduce the characteristics of the data. We performed experiments and compared the performance of XGBoost in terms of single sequence feature information and spliced sequence feature information. On the PDB 1075 data set, performance of the spliced sequence feature (MCC: 0.7272) is obviously better than that of the single sequence feature. To further assess our method, we applied the XGBoost model to the PDB186 and PDB2272 data sets. XGBoost produced superior results for PDB186 (MCC: 0.713) and PDB2272 (MCC: 0.5652) compared to available methods.

## Data Availability

The original contributions presented in the study are included in the article/supplementary material. Further inquiries can be directed to the corresponding authors.
